# Cell surface vimentin: a natural human immune response target for immunotherapy

**DOI:** 10.3389/fmmed.2025.1552323

**Published:** 2025-02-20

**Authors:** Mark C. Glassy

**Affiliations:** Neuro-Oncology Laboratory, Moores Cancer Center, University of California, San Diego, San Diego, CA, United States

**Keywords:** vimentin, pritumumab, immunotherapy, antibody cocktails, lymph nodes, germinal centers

## Abstract

Natural human monoclonal antibodies obtained from sentinel lymph nodes of cancer patients identify cell surface vimentin. One of these vimentin-reactive antibodies, pritumumab, has been used to treat brain cancer patients. This review summarizes data on mAbs reactive with cell surface vimentin and their origin from lymph nodes of cancer patients.

## Introduction

Intermediate filaments are a class of dynamic, flexible cytoplasmic proteins such as keratin, vimentin, desmin, and lamin (Dutour-Provenzano and Etienne-Manneville, 2021; Satelli and Li, 2011). Vimentin in involved in cell processes, including cell migration, cell shape and plasticity, and organelle anchorage (Mendez et al., 2010; Mendez et al., 2014; Ivaska et al., 2007; Eckes et al., 1998; Ebert et al., 2000), possibly by interacting with actin (Esue et al., 2006). Vimentin is also implicated in several pathophysiological conditions such as cancer (van Loon et al., 2024), autoimmune and inflammatory diseases, and infection (Kidd et al., 2014; Lazarova and Bordonaro, 2016; Brzozowa et al., 2015; Zhao et al., 2013; Yin et al., 2018; Peng et al., 2022), including wound healing (Eckes et al., 2000) and evading immune surveillance (Peng et al., 2022). Vimentin is expressed in many cell types including mesenchymal cells, fibroblasts, endothelial cells, macrophages, melanocytes, Schwann cells, and lymphocytes (Dutour-Provenzano and Etienne-Manneville, 2021; Satelli and Li, 2011). In addition to its cytoplasmic location vimentin can also be expressed at the cell surface (Bhattacharya et al., 2009; Heming et al., 2024; Tabatabaee et al., 2024) and that its role as a cell surface marker in oncology is mostly unknown.

The recognition of cell surface vimentin suggests an innate auto-antigenic natural human immune response (Glassy, 2020), perhaps a function of immunosurveillance (Peng et al., 2022). It follows then that if cell surface vimentin were not present then no anti-vimentin response would occur. On the other hand, when cells lyse and release cytoplasmic components, one of which is vimentin, then perhaps the natural human immune response has a pre-established response to vimentin and when it appears on the cell surface this natural pre-established response generates natural antibodies to vimentin.

Since natural antibodies to vimentin exist (Glassy, 2020; Nasoff et al., 1997) then where are the B-cells that make them? One potential answer is the lymph node (LN). Therefore, how does vimentin–and what form of vimentin - gets into LNs to stimulate such an immune response? Is vimentin by itself, an altered form, or is it complexed with other biomolecules as it enters the LN? Natural immune cell processing of vimentin, such as HLA recognition and dendritic cell activation, occurs so are any vimentin epitopes ‘more immunogenic’ than other epitopes? What role does post-translational epigenetic processes play in focusing a human response on a particular vimentin epitope?

Epithelial–mesenchymal transition (EMT) is a reversible cellular program that transiently places epithelial cells into quasi-mesenchymal cell states (Kalluri and Weinberg, 2009; Dongre and Weinberg, 2019; Nieto, 2009). During the EMT process there is an upheaval in DNA expressions in that gene expression increases and decreases, some even superinduced (Dongre and Weinberg, 2019). During this process the activation of EMT mechanisms results in the progressive loss of the typical polygonal, cobblestone morphology of epithelial cells into that of spindle-shaped, mesenchymal morphology (Dongre and Weinberg, 2019). These cells express markers that are associated with the mesenchymal cell state, notably neural cadherin (N-cadherin), vimentin and fibronectin (Arrindell and Desnues, 2023).

## Pritumumab

Pritumumab, a natural human IgG1 kappa antibody, was obtained from a regional draining lymph node of a patient with cervical carcinoma through traditional hybridoma technology (Glassy et al., 1983; Glassy and Gupta, 2013). Specificity analysis of the target antigen, an altered form of vimentin called, ecto-domain vimentin (EDV), shows it to be limited to cell surface expression on cancer cells (Glassy, 2020; Babic et al., 2019). However, it is currently unclear what structural modifications resulted in EDV and its role, either directly or indirectly, in tumor biology. In early clinical trials with hybridoma-generated pritumumab 249 brain cancer patients were treated with a low dose regimen, either at 1 mg once a week or 1 mg twice a week, and of those evaluated their overall response rates of between 25% and 30% were seen with several complete and partial responses (Glassy and Hagiwara, 2009).

A second Phase 1 trial with was completed with CHO-produced pritumumab (Gupta et al., 2013) that also showed clinical benefit (Carrillo et al., 2024). Overall, 15 patients received pritumumab in a recurrent setting. Pritumumab was well tolerated, with no serious adverse events related to the treatment reported. The most common pritumumab-related toxicities were constipation and fatigue. There were no dose-limiting toxicities observed, and a maximum tolerated dose was not reached. Thus, the maximum feasible dose and recommended phase 2 dose of pritumumab was established at 16.2 mg/kg weekly. Out of eleven patients evaluated for efficacy, one patient (9.1%) demonstrated partial response based on response assessment in neuro-oncology criteria, and disease stabilization was seen in 3 patients (27.3%). This data suggests that the CHO-produced pritumumab is well tolerated with no dose limiting toxicities observed up to 16.2 mg/kg weekly (Carrillo et al., 2024). Overall, these data together suggests vimentin-targeted pritumumab is suitable for further development as an anti-tumor therapeutic.

## RM2

**FIGURE 1:**
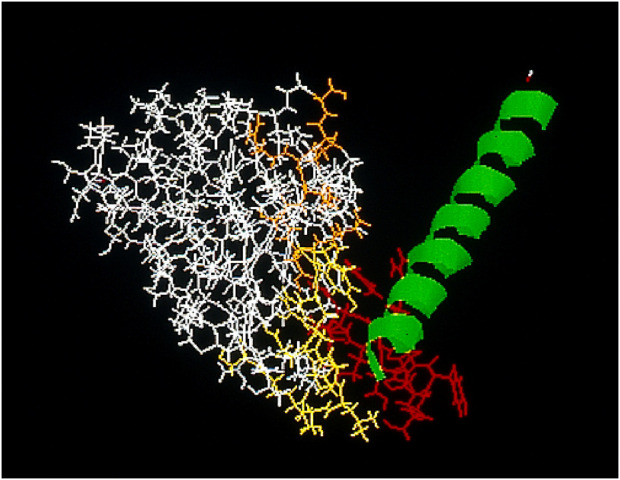
In silico analysis of CDR sequence of RM2, yellow, and orange, against the IGK4 peptide sequence of vimentin, green, which contains the reactive epitope (red).

Regional draining lymph nodes from patients with colon and pancreatic cancers were obtained from surgical specimens at biopsy, pooled, processed under sterile conditions, and then stimulated *in vitro* with pokeweed mitogen [PWM; Koda and Glassy (1990)] to enhance cell proliferation. Using standard hybridoma procedures RM2, a natural human IgG, was obtained (Nasoff et al., 1997; Glassy et al., 2007). FACS analysis showed the RM2 antigen to be cell surface expressed (Nasoff et al., 1997; Glassy et al., 2007) and standard Western blot analysis of RM2 binding showed a single chain protein antigen with an apparent molecular weight of 52 kDa (Nasoff et al., 1997). Subsequent analysis has shown the antigen to be an epitope on the intermediate filament vimentin (Babic et al., 2019). In silico data showed the IGK4 sequence of the coil 2B rod fragment of vimentin binds to RM2 [Figure 1; Culler et al. (2004)].

Additional studies showed that RM2 has a similar immunohistological binding pattern as pritumumab (Glassy and Hagiwara, 2009; Glassy et al., 2007) and, like pritumumab, is also effective in reducing tumor burdens in xenograft models (Glassy et al., 2007).

## EDV as a biomarker

Based on the selective immunoreactivity of cell surface vimentin, with both pritumumab and RM2, it may be useful as a biomarker since it is not shed nor internalized upon mAb binding (Glassy, 2020; Glassy and Hagiwara, 2009). Therefore, as a mechanism of action the mAb does not have to penetrate cells as in the case with immunoconjugates (Anubhab et al., 2019). Immunohistological evaluation showed that EDV is present on all three germ layers, ectoderm, endoderm, and mesoderm, and therefore may be a tumor-restricted pan-cancer antigen (Glassy, 2020; Glassy and Hagiwara, 2009). EDV is involved in EMT transitions and could therefore be considered a neo-antigen in which the reactive epitopes may be epigenetically derived. Both slow and rapidly growing cells are EDV + so circulating tumor cells may have diagnostic utility.

Cancer stem cells that are CD144+ are also EDV + so immunotherapeutic protocols based on EDV reactivity may also be effective and show a response with early cancer cell development. CD144+ cancer stem cells are precursors to tumor development and by targeting EDV on these cells may have implications in mitigating cancer progression.

## Natural heavy chain sequences

Current mAb oncology targets have been identified by murine xenogenic responses (Kothari et al., 2024). The Complimentary Determining Region (CDR) sequences from these murine mAbs have been incorporated into human IgG scaffolds resulting in humanized antibodies (Donzeau and Knappik, 2007). Something that has not been discussed in detail is the generic heavy chain sequences of these humanized mAbs, which are essentially all the same, and all derivative of the same sequence. These heavy chain sequences may be too uniform to fully integrate into the natural human humoral immune response. There are minor differences in human IgG heavy chain sequences that may provide for more efficient effector functions. Selecting a natural IgG heavy chain scaffold may be a more effective tool for a more robust immune response than the generic IgG sequences used in generating recombinant forms for production (Donzeau and Knappik, 2007). The IgG heavy chain sequences of pritumumab and RM2 are natural, not generic, with no modifications. In the germinal center of the original patients, whose lymph nodes were removed resulting in the identification of pritumumab as well as RM2, the natural affinity matured CDR sequences were combined with the natural isotype (IgG1kappa) the patient generated in the original immune response to the target, ecto-domain vimentin.

## Vimentin cocktail utility

A natural application of antibodies binding to the same antigen is by combining the mAbs into a cocktail which may more accurately reflect the natural human oligoclonal response (Gupta and Glassy, 2014; Glassy et al., 2007). Since the natural immune response is oligoclonal then more than one antibody to a given target is how natural immunity works. RM2 and pritumumab could be combined into a Multimab cocktail with binding different co-expressed epitopes on the same antigen on the same cell (Mukerjee et al., 1999). This may result in a more robust immune response. Antibodies in general act in a synergistic way (Glassy et al., 2007) and a vimentin Multimab cocktail may enhance such an immune response.

## Neoantigen possibilities

As a result of the EMT transition the neo-epitopes that appear on vimentin are most likely due to epigenetic modifications since both normal and EDV are expressed by the same cancer cell which suggests the gene coding for vimentin is unchanged and not due to alternate splicing variants. Further work will be necessary to determine which genetic changes cause these neo-epitope modifications to appear.

## Lymph nodes

Since both pritumumab and RM2 are lymph node derived suggests LNs are a valuable source for onco-important targets (Node Biopsy and Ioachim, 1982; Glassy and McKnight, 1994; Okadome et al., 1991; Yagyu et al., 1992; Tanigawa et al., 2001). LNs are among the organs most commonly used for staging for biopsy and diagnosis (Glassy and McKnight, 1994). They are serviced by the lymphatic pathway, both afferent and efferent lymphatics, and the blood circulatory system. The focus in LNs are the germinal centers, the sites for antibody class switching and affinity maturation (Victoria and Nussenzweig, 2022). In oncology LNs are important because metastatic cells migrate through them (Fares et al., 2020). The intelligence of the immune response of sentinel lymph nodes in cancer can be exploited by analyzing antibodies derived from these germinal centers (Yagyu et al., 1992; Glassy, 1987). Both RM2 and pritumumab are germinal center-derived.

Germinal centers vary in size, increasing dramatically with antigen challenge. The lymphoid cells in germinal centers consist of small, medium-sized, and large lymphocytes in various degrees of maturation, stimulation, and proliferation. An analysis of the antibody repertoire of these B cells could be useful in understanding the limitations and broadness of natural antibodies to tumor antigens.

In the germinal centers of cancer patients major V-D-J gene rearrangements have occurred whereby antibodies to various tumor associated antigens have been generated. In this way, the intelligence of the natural immune response has acted like a drug discovery program in which the human immune response has, in essence, identified, located, and responded to tumor antigens (Glassy and McKnight, 1993). This lymph node response, that is, germinal center development, is antigen driven. Overall, immunohistochemical analysis has shown these lymph node derived human antibodies recognize antigens which are highly restricted to tumor cells and tissues (Glassy et al., 2007; Glassy, 1987).

Migrating lymphocytes filter through lymph nodes and, if necessary, stay and develop into germinal centers through clonal selection and expansion (Fares et al., 2020). Affinity maturation may occur at this time as well as class switching, which is cytokine driven. Specificity remains the same though the heavy chains and perhaps affinity change. All these responses are antigen driven and therefore constitute “antigen-specific modulation(s)”.

This then begs the questions of what is the nature of the antigen and how does it drive somatic hypermutation (v-gene editing) and affinity maturation in LNs? It is noted that antigen must constantly be present to keep driving germinal center development (Victoria and Nussenzweig, 2022). Is the percent time spent in the presence of antigen related to affinity maturation? One possibility is antigen processing could occur outside of the LN, such as seen with tumor infiltrating lymphocytes (Kotlan et al., 2003; Kotlan et al., 2006), then “armed” dendritic cells enter the LN for germinal center development (Victoria and Nussenzweig, 2022).

In a normal, unreactive lymph node there are about 20 germinal centers. In a reactive lymph node there can be up to 100 germinal centers. Average germinal center size is ∼0.1 cm in length and ∼0.001 cm^3^. Approximate normal LN size is 0.6 × 0.3 × 0.3 cm = 0.054 cm^3^. A reactive LN is: 1.2 × 0.8 × 0.6 = 0.57 cm^3^. There are about 6 × 10^8^ lymphocytes in each unreactive LN and about 6.5 × 10^9^ cells in a reactive LN. Many of these lymphocytes reflect clonal expansion (Victoria and Nussenzweig, 2022).

Since humans do make anti-cancer antibodies then what is the germinal center antibody repertoire and what predictions can we make from this? What can we learn about the natural human immune response from these patients? Can any insight be gained in analyzing the theoretical limits of the regional draining lymph node immune response? Are there any general rules in sentinel germinal center development based on the antibody repertoire?

LNs are connected by lymphatic vessels so their echelon from proximal to distal may reflect a different anti-cancer response (Victoria and Nussenzweig, 2022). The antibody repertoire of proximal, sentinel LNs may be different from distal LNs (Cochran et al., 1992; Morton et al., 1992). Does this antibody repertoire vary between different cancers? Since it appears EDV may be a pan-cancer surface target then processing the antigen by germinal centers could be considered a common process irrespective of the type of epithelial cancer.

In our overall analysis of antibodies obtained from regional draining lymph nodes of cancer patients suggests the recognized antigens consist of various cell surface proteins including gangliosides (Mukerjee et al., 1998; Kotlan et al., 2005). Animal models of biodistribution and tumor regression with these natural mAbs suggests they have bioactivity in immunotargeting and immunoregulating cancer (Glassy et al., 2007; Koda et al., 1998a).

Clinical data from phase I/II trials with cancer patients suggests these LN-derived mAbs show patient benefits (Glassy, 2020; Glassy and Hagiwara, 2009; Carrillo et al., 2024). Since these natural human antibodies have all been obtained from reactive lymph nodes of cancer patients suggests that interesting V-D-J antibody gene rearrangements have occurred, most likely driven by exposure to various tumor antigens, such as EDV. Such a panel of natural LN-derived human antibodies, formulated as a cocktail, may have utility in the oncology clinic (Glassy et al., 2007; Glassy and McKnight, 2005; Krieg et al., 2022). Afterall, the natural human immune response is oligoclonal so a few antibodies most likely are generated to each target antigen (Gupta and Glassy, 2014).

Since LNs appear to be an interesting source of anti-cancer mAbs (Glassy, 1987; Koda et al., 1990; Koda et al., 1998b; Koda et al., 2001) then one option is to generate a natural lymph node CDR library from these antibodies. Patterns in CDR profiles, such as families and sub-families may provide insight into the natural anti-cancer immune response. Construction of CDR libraries have provided insight as to the feasibility of this approach (Kotlan et al., 2003). Each sentinel LN in cancer patients is an immunological snapshot of an anti-cancer response (Kothari et al., 2024; Glassy and McKnight, 1993). Can LN-derived onco-relevant CDR sequences be organized into a “tree of life” to better understand the oncogenic process?

Questions difficult to answer: What type of antigen, soluble or cell-bound, is best for generating an anti-cancer lymph node germinal center immune response? Furthermore, what is the antigen threshold for generating an immune response? How effective is the germinal center response in generating the most appropriate heavy chain as well as the optimal affinity? What sort of oligoclonal response can be generated by such involved nodes?

A major advantage of exploiting the LN human antibody repertoire is the ability to re-introduce a natural antibody back into patients that should be well tolerated. After all, human IVIG preparations can use up to 50 g per treatment (Schwab and Nimmerjahn, 2013) so a much smaller dose of a natural human antibody should be well tolerated.

What sort of anti-tumor response occurs in nodes which are microscopic only in extent and not detectable grossly? The location of the LN, proximal to distal, in relation to the primary tumor is important. Micro metastatic foci in LNs may trigger an immune response as measured by germinal center development (Victoria and Nussenzweig, 2022). There may be microheterogeneity with multiple metastasis that could affect germinal center development. The size of these foci may be important in presenting antigen load. An important question to ask is when does “occult” foci turn into “non-occult” foci?

Tumor cells have an advantage in rapid growth (fast cell cycle times) so they can both stimulate an immune response and “outgrow” the insufficient retaliation. Tumor cell doubling time is critical here. The natural immune response may not be sufficient to keep up with rapidly growing tumor cells so tumor antigen levels are in a higher concentration than antibodies that can be generated.

Some predictive elements of regional draining LNs (Victoria and Nussenzweig, 2022) consist of the size (occult vs. gross), number and location such as sentinel or secondary level, ipsilateral or bilateral, the extent of nodal invasion, edema, pericapsular sinus or replacement, including extracapsular, vascular, lymphatic, neural, or soft tissue invasion. In addition is the growth potential of metastases within the LN.

Analysis of the B cell immune response from sentinel LNs could provide information on the immunological fitness of the antigen. Sentinel LNs are a vibrant resource which provides an interesting window from which to observe the natural anti-cancer immune response. It is also important to point out that in sentinel LNs some cancer antigens may be more immunogenic than others, some dormant and others more active, and patient’s immune responses may reflect this. Some antigens may have a Jekyll/Hyde function in which dormant antigens can be made Hyde-like immunogenic and *vice versa*, Hyde to Jekyll, to escape from the immune response. Since humans do make antibodies to their own cancer antigens then how can this be best exploited to benefit patients?

## Summary

Cell surface vimentin is ready for immunotherapy primetime. With the identification of two separate immune responses in cancer patients to vimentin, pritumumab and RM2, all derived from sentinel LNs, strongly suggests that there may be other patients that also mount an immune response to cell surface vimentin (EDV). This natural immune response to EDV may be oligoclonal and a few antibodies are generated to vimentin, perhaps to different epitopes. The most obvious application of such an oligoclonal response is targeting surface vimentin with a cocktail of human mAbs with each mAb to a different epitope. Antibody cocktails have been shown to be more effective than monotherapy and such cocktails may reflect a natural oligoclonal oncology response (Gupta and Glassy, 2014; Glassy et al., 2007; Schwab and Nimmerjahn, 2013).

RM2 and pritumumab bind to vimentin, the same antigen but a different epitope, and both came from a different lymph node which strongly suggests the target, vimentin, is onco-important irrespective of the origin of the cancer type. Also, the immunohistological data suggests all forms of solid tumors of epithelial origin are EDV+. Irrespective of the tumor type it appears LNs may process epithelial tumor cells in such a way that surface vimentin (EDV) is a recognized target. Peptide sequences of vimentin have been generated (Hagiwara et al., 2001) and unfolded peptides of the binding region bind to pritumumab (by Western blot) whereas alpha-helical peptides do not bind to pritumumab (M. Pellecchia & Glassy, unpublished data) which suggests the epitope is conformation dependent.

An interesting aspect about pritumumab is its ability to cross the blood-brain barrier (BBB). Implanted orthotopic brain tumors in mice were successfully imaged within 4 hours suggesting the antibody crossed the BBB (Modi et al., 2024). One possible mechanism involved the antibody’s isoelectric point of 8.6. A high isoelectric point may contribute to the rapid passage through the BBB (Modi et al., 2024).

The cytoskeleton gets remodeled in cancer and vimentin is one of these remodeled proteins as a result of the EMT events (Dongre and Weinberg, 2019). Vimentin seems to have a “Jekyll/Hyde” aspect. Internal cytoplasmic or Jekyll vimentin acts normally whereas cell surface Hyde vimentin reflects oncology circumstances with a remodeled cytoskeleton.

Nature does not work in a vacuum so if two independent studies have yielded mAbs to the same target, vimentin, then there must be others. The question is how frequent is the immune response to vimentin? Also, do these anti-vimentin antibodies have any implication in the anti-cancer immune response? One possible interpretation is the natural immune response to EDV does cause cell death resulting in necrotic lesions but the growth of the cancer cells outpaces the production of effective antibodies. From this one can then ask how many LN-derived antibodies are cell specific? Are these identified antigens cell surface, cytoplasmic, or even secreted?

LNs of cancer patients may represent a model system to probe antigen driven immunoselection of antibody-secreting anti-cancer B cells, which occurs in germinal centers, where affinity maturation, class switching, and somatic hypermutation events take place. Overall, the data suggests that interesting LN antibody responses are generated to a class of antigens which impact tumor biology. Since tumors do grow then other elements (e.g., cytotoxic T cells, various cytokines, etc.) are necessary to eradicate and/or control tumor cell development. Growth of cancer cells may outpace the production and bioavailability of an effective immune response.

Future research directions should include an understanding of how the processing of vimentin to EDV influences malignancy. Is EDV directly or indirectly involved in tumor progression or perhaps the tumor microenvironment? Also, what is the precise epigenetic modification(s) used to separate normal cytoplasmic vimentin from cell surface vimentin? It is unclear what, if any, specific biological function EDV has. Is EDV alone on the cell surface or is it complexed with other biomolecules?

A sentinel LN processed vimentin to generate an antibody response which, in turn, recognizes a form of vimentin located on the cell surface of cancer cells. This antibody may have utility in the treatment of epithelial cancer cells that are cell surface vimentin positive. The data suggests vimentin should be included as a viable marker and potential target in oncology.

## References

[B1] Anubhab M. Watersa A. K. Babic I. Nurmemmedov E. Glassy M. C. Kesari S. (2019). “Antibody drug conjugates: progress, pitfalls, and promises,” Hum. Antibodies, 27. 53–62. 10.3233/HAB-180348 30223393

[B2] Arrindell J. Desnues B. (2023). Vimentin: from a cytoskeletal protein to a critical modulator of immune response and a target for infection. Front. Immunol. 14, 1224352. 10.3389/fimmu.2023.1224352 37475865 PMC10354447

[B3] Babic I. Kesari S. Glassy M. C. (2019). “A binding potency assay for pritumumab and ecto-domain vimentin,” in Methods in molecular biology; vol 1904; human monoclonal antibodies; methods and protocols. (Clifton, N.J: Humana Press). 10.1007/978-1-4939-8958-4_19 30539482

[B4] Bhattacharya R. Gonzalez A. M. DeBiase P. J. Trejo H. E. Goldman R. D. Flitney F. W. (2009). Recruitment of vimentin to the cell surface by beta3 integrin and plectin mediates adhesion strength. J. Cell Sci. 122, 1390–1400. 10.1242/jcs.043042 19366731 PMC2721003

[B5] Brzozowa M. Wyrobiec G. Kolodziej I. Sitarski M. Matysiak N. Reichman- Warmusz E. (2015). The aberrant overexpression of vimentin is linked to a more aggressive status in tumours of the gastrointestinal tract. Prz. Gastroenterol. 10, 7–11. 10.5114/pg.2014.47502 25960808 PMC4411408

[B6] Carrillo J. Gill J. M. Redfern C. Babic I. Nomura N. Shah D. K. (2024). A phase 1 dose escalation of pritumumab in patients with refractory or recurrent gliomas or brain metastases. Neuro-Oncology Adv. 6 (1), vdae166. 10.1093/noajnl/vdae166 PMC11502913 39465217

[B7] Cochran A. J. Wen D.-R. Morton D. L. (1992). Management of the regional lymph nodes in patients with cutaneous malignant melanoma. World J. Surg. 16, 214–221. 10.1007/BF02071523 1561801

[B8] Culler S. Hsaio T. Glassy M. Chau P. (2004). Cluster and information entropy analysis of the complementarity determining regions in antibodies. BioSystems 77, 195–212. 15527957 10.1016/j.biosystems.2004.05.033

[B9] Dongre A. Weinberg R. A. (2019). New insights into the mechanisms of epithelial-mesenchymal transition and implications for cancer. Nat. Rev. 20, 69–84. 10.1038/s41580-018-0080-4 30459476

[B10] Donzeau M. Knappik A. (2007). Recombinant monoclonal antibodies. Methods Mol. Biol. 378, 14–31. 10.1007/978-1-59745-323-3_2 18605075

[B11] Dutour-Provenzano G. Etienne-Manneville S. (2021). Intermediate filaments. Curr. Biol. 31, R522–R529. 10.1016/j.cub.2021.04.011 34033784

[B12] Ebert A. D. Wechselberger C. Nees M. Clair T. Schaller G. Martinez-Lacaci I. (2000). Crypto-1-induced increase in vimentin expression is associated with enhanced migration of human caski cervical carcinoma cells. Exp. Cell Res. 257, 223–229. 10.1006/excr.2000.4881 10854071

[B13] Eckes B. Colucci-Guyon E. Smola H. Nodder S. Babinet C. Krieg T. (2000). Impaired wound healing in embryonic and adult mice lacking vimentin. J. Cell Sci. 113, 2455–2462. 10.1242/jcs.113.13.2455 10852824

[B14] Eckes B. Dogic D. Colucci-Guyon E. Wang N. Maniotis A. Ingber D. (1998). Impaired mechanical stability, migration and contractile capacity in vimentin-deficient fibroblasts. J. Cell Sci. 111, 1897–1907. 10.1242/jcs.111.13.1897 9625752

[B15] Esue O. Carson A. A. Tseng Y. Wirtz D. (2006). A direct interaction between actin and vimentin filaments mediated by the tail domain of vimentin. J. Biol. Chem. 281, 4130393–4130399. 10.1074/jbc.m605452200 16901892

[B16] Fares J. Fares M. Y. Khachfe H. H. Salhab H. A. Fares Y. (2020). Molecular principles of metastasis: a hallmark of cancer revisited. Sig Transduct. Target Ther. 5, 28. 10.1038/s41392-020-0134-x PMC7067809 32296047

[B17] Glassy M. McKnight M. (2005). Requirements for human antibody cocktails for oncology. Expert. Opin. Biol. Ther. 5, 1333–1338. 10.1517/14712598.5.10.1333 16197338

[B18] Glassy M. McKnight M. Kotlan B. Glassy E. Koda K. (2007). Cocktails of human anti-cancer antibodies show a synergistic effect in nude mouse tumor xenografts. Hum. Antibod 16, 87–98. 10.3233/hab-2007-163-403 18334744

[B19] Glassy M. C. (1987). Immortalization of human lymphocytes from a tumor involved lymph node. Cancer Res. 47, 5181–5188. 3621203

[B20] Glassy M. C. (2020). Unconventional immunotherapy with an unconventional target. Hum. Antibod, 1–6. 10.3233/HAB-200427 32925025

[B21] Glassy M. C. Gupta R. (2013). Technical and ethical limitations in making human monoclonal antibodies (an overview). Methods Mol. Biol. Clifton, N.J. 1060, 9–36. 10.1007/978-1-62703-586-6_2 24037834

[B22] Glassy M. C. Hagiwara H. (2009). Summary analysis of the pre-clinical and clinical results of brain tumor patients treated with pritumumab. Hum. Antibod 18, 127–137. 10.3233/HAB-2009-0209 19996527

[B23] Glassy M. C. Handley H. H. Hagiwara H. Royston I. (1983). UC 729-6, a human lymphoblastoid B cell line useful for generating antibody secreting human-human hybridomas. Proc. Natl. Acad. Sci. U. S. A. 80, 6327–6331. 10.1073/pnas.80.20.6327 6604917 PMC394290

[B24] Glassy M. C. McKnight M. E. (1993). A novel drug discovery program utilizing the human immune response. Curr. Opin. Invest. Drugs 2, 853–858.

[B25] Glassy M. C. McKnight M. E. (1994). Pharming the human lymph node. Exp. Opin. Invest. Drugs 3, 1057–1060. 10.1517/13543784.3.10.1057

[B26] Gupta R. Glassy M. C. (2014). “Oligoclonal and polyclonal antibodies in immunotherapy,” in Handbook of immunotherapy.

[B27] Gupta R. York D. Kotlan B. Bleck G. Glassy E. Glassy M. (2013). Use of the Gpex® system to increase production of pritumumab in a CHO cell line. J. Bioprocess Technol. Phot. 98, 318–326.

[B28] Hagiwara H. Yasuyuki A. Yasushi Y. Junichi M. Yuko M. (2001). Determination of the antigen/epitope that is recognized by human monoclonal antibody CLN-IgG. Hum. Antibod 10, 77–82. 11673662

[B29] Heming L. Xu L. Zhao M. Han T. Luan J. (2024). Exploring new frontiers: cell surface vimentin as an emerging marker for circulating tumor cells and a promising therapeutic target in advanced gastric Cancer. J. Exp. Clin. Cancer Res. 43 (1), 129. 10.1186/s13046-024-03043-6 38685125 PMC11059585

[B30] Ivaska J. Pallari H. M. Nevo J. Eriksson J. E. (2007). Novel functions of vimentin in cell adhesion, migration, and signaling. Exp. Cell Res. 313, 2050–2062. 10.1016/j.yexcr.2007.03.040 17512929

[B31] Kalluri R. Weinberg R. A. (2009). The basics of epithelial–mesenchymal transition. J. Clin. Invest. 119, 1420–1428. 10.1172/JCI39104 19487818 PMC2689101

[B32] Kidd M. E. Shumaker D. K. Ridge K. M. (2014). The role of vimentin intermediate filaments in the progression of lung cancer. Am. J. Respir. Cell Mol. Biol. 50, 1–6. 10.1165/rcmb.2013-0314TR 23980547 PMC3930939

[B33] Koda K. Glassy M. McKnight M. Saito N. Dan M. Fukao K. (1998b). Nakajima, Human monoclonal antibody SK-1 immunotargeting for rectal carcinoma. N. Intl. J. Immunother. 14, 153–161.

[B34] Koda K. Glassy M. C. (1990). *In vitro* immunization for the production of human monoclonal antibody. Hum. Antibod. Hybridomas 1, 15–22. 10.3233/hab-1990-1104 2103349

[B35] Koda K. Glassy M. C. Chang H. R. (1990). Generation of human monoclonal antibodies against colon cancer. Arch. Surg. 125, 1591–1597. 10.1001/archsurg.1990.01410240073015 2244813

[B36] Koda K. Glassy M. C. McKnight M. E. Yasutomi J. Saito N. Dan M. (2001). Immunotherapy for recurrent colorectal cancers with human monoclonal antibody SK1. Anticancer Res. 21, 621–627. 11299816

[B37] Koda K. Nakajima N. Saito N. Yasutomi J. McKnight M. E. Glassy M. C. (1998a). A human natural antibody to adenocarcinoma that inhibits tumour cell migration. Brit. J. Cancer 78, 1313–1322. 10.1038/bjc.1998.677 9823972 PMC2063195

[B38] Kothari M. Wanjari A. Acharya S. Karwa V. Chavhan R. Kumar S. (2024). A comprehensive review of monoclonal antibodies in modern medicine: tracing the evolution of a revolutionary therapeutic approach. Cureus 16 (6), e61983. 10.7759/cureus.61983 38983999 PMC11231668

[B39] Kotlan B. Simsa P. Foldi J. Fridman W. H. Glassy M. McKnight M. (2003). Immunoglobulin repertoire of B lymphocytes infiltrating breast medullary carcinoma. Hum. Antibod 12, 113–121. 10.3233/hab-2003-12402 15156099

[B40] Kotlan B. Simsa P. Teillaud J. L. Fridman W. F. Toth J. McKnight M. (2005). Novel ganglioside antigen identified by B cells in human medullary breast carcinomas: the proof of principle concerning the tumor-infiltrating B lymphocytes. J. Immunol. 175, 2278–2285. 10.4049/jimmunol.175.4.2278 16081796

[B41] Kotlan B. Toth J. McKnight M. Glassy M. C. (2006). Characteristics of tumor gangliosides revealed by B cells infiltrating human breast carcinomas. Hum. Antibod 15, 9–12.

[B42] Krieg D. Winter G. Svilenov H. L. (2022). It is never too late for a cocktail - development and analytical characterization of fixed-dose antibody combinations. J. Pharmeceut. Sci. 111, 2149–2157. 10.1016/j.xphs.2022.05.014 35598781

[B43] Lazarova D. L. Bordonaro M. (2016). Vimentin, colon cancer progression and resistance to butyrate and other HDACis. J. Cell Mol. Med. 20, 989–993. 10.1111/jcmm.12850 27072512 PMC4882977

[B44] Mendez M. Restle D. Janmey P. (2014). Vimentin enhances cell elastic behavior and protects against compressive stress. Biophys. J. 107, 314–323. 10.1016/j.bpj.2014.04.050 25028873 PMC4104054

[B45] Mendez M. G. Kojima S. Goldman R. D. (2010). Vimentin induces changes in cell shape, motility, and adhesion during the epithelial to mesenchymal transition. FASEB J. 24, 1838–1851. 10.1096/fj.09-151639 20097873 PMC2874471

[B46] Modi A. Wang F. Mukthavaram R. Jiang P. Gangangari K. Pillarsetty N. (2024). Preclinical characterization of CHO-derived pritumumab targeting ecto-domain vimentin in xenograft models and primate safety. Sci. Rep. 10.1038/s41598-025-95360-9 PMC11933471 40128557

[B47] Morton D. L. Wen D.-R. Wong J. H. Economou J. S. Cagle L. A. Storm F. K. (1992). Technical details of intraoperative lymphatic mapping for early stage melanoma. Arch. Surg. 127, 392–399. 10.1001/archsurg.1992.01420040034005 1558490

[B48] Mukerjee S. McKnight M. E. Nasoff M. Glassy M. C. (1999). “Co-expression of tumor antigens and their modulation by pleiotrophic modifiers enhance targeting of human monoclonal antibodies to pancreatic carcinoma,” Hum. Antibodies, 9. 9–22. 10.3233/hab-1999-9102 10331182

[B49] Mukerjee S. Nasoff M. McKnight M. Glassy M. C. (1998). Characterization of human IgG1 monoclonal antibody against gangliosides expressed on tumor cells. Hybridoma 17, 133–142. 10.1089/hyb.1998.17.133 9627053

[B50] Nasoff M. Gu M. Galindo J. He X.-M. Mukerjee S. McKnight M. (1997). Cloning and expression of the human tumor specific antibody GM4. Hybridoma 16, 427–439. 10.1089/hyb.1997.16.427 9388026

[B51] Nieto M. A. (2009). Epithelial-Mesenchymal Transitions in development and disease: old views and new perspectives. Int. J. Dev. Biol. 53, 1541–1547. 10.1387/ijdb.072410mn 19247945

[B52] Node Biopsy L. Ioachim H. L. (1982). Philadelphia: Lippincott Pub.

[B53] Okadome M. Saito T. Tsukamoto N. Sano M. Kamura T. Nakano H. (1991). Potential of human lymph node cells for antitumor activity mediated by interferon gamma. Cancer 68, 2378–2383. 10.1002/1097-0142(19911201)68:11<2378::aid-cncr2820681108>3.0.co;2-2 1933774

[B54] Peng J.-M. Chiu C.-F. Cheng J.-H. Liu H.-Y. Chang Y.-L. Luo J.-W. (2022). Evasion of NK cell immune surveillance via the vimentin-mediated cytoskeleton remodeling. Front. Immunol. 13, 883178. 10.3389/fimmu.2022.883178 36032170 PMC9402923

[B55] Satelli A. Li S. (2011). Vimentin in cancer and its potential as a molecular target for cancer therapy. Cell Mol. Life Sci. 68, 3033–3046. 10.1007/s00018-011-0735-1 21637948 PMC3162105

[B56] Schwab I. Nimmerjahn F. (2013). Intravenous immunoglobulin therapy: how does IgG modulate the immune system? Nat. Rev. Immunol. 13, 176–189. 10.1038/nri3401 23411799

[B57] Tabatabaee A. Nafari B. Farhang A. Mirian M. (2024). Extracellular vimentin as a versatile immune suppressive protein in cancer**.** September 2023 Biochim Biophys Acta. Rev. Cancer. 10.1016/j.bbcan.2023.188985 37717859

[B58] Tanigawa K. Takeshita N. Craig R. A. Phillips K. Knibbs R. N. Chang A. E. (2001). Tumor-specific responses in lymph nodes draining murine sarcomas are concentrated in cells expressing P-selectin binding sites. J. Immunol. 167, 3089–3098. 10.4049/jimmunol.167.6.3089 11544293

[B59] van Loon K. van Breest Smallenburg M. E. Huijbers E. J. M. van Beijnum J. R. (2024). Targeting vimentin: a multifaceted approach to combatting cancer metastasis and drug resistance. Cancer Metastasis Rev. 10.1007/s10555-023-10154-7 38012357

[B60] Victoria G. D. Nussenzweig M. C. (2022). Germinal centers. Ann. Rev. Immunol. 40, 413–442. 10.1146/annurev-immunol-120419-022408 35113731

[B61] Yagyu T. Monden T. Tamaki Y. Morimoto H. Takeda T. Kobayashi T. (1992). Use of a local immunotherapy as an adjunctive tool for the generation of human monoclonal antibodies from regional lymph nodes of colonic cancer patients. J. Cancer Res. 83, 20–23. 10.1111/j.1349-7006.1992.tb02346.x PMC5918647 1544869

[B62] Yin S. Chen F. F. Yang G. F. (2018). Vimentin immunohistochemical expression as a prognostic factor in gastric cancer: a meta-analysis. Pathol. Res. Pract. 214, 1376–1380. 10.1016/j.prp.2018.07.014 30078472

[B63] Zhao W. Yue L. Zhou F. Xu C. Liang W. Sui A. (2013). Clinical significance of vimentin expression and her-2 status in patients with gastric carcinoma. Clin. Transl. Sci. 6, 184–190. 10.1111/cts.12043 23751022 PMC5350850

